# How the Renal Tubule May Adapt to Fuel Shortage

**DOI:** 10.34067/KID.0000000826

**Published:** 2025-05-29

**Authors:** Verónica Miguel, Santiago Lamas

**Affiliations:** 1 Spanish National Center for Cardiovascular Research (CNIC), Madrid, Spain; 2 Program of Physiological and Pathological Processes, Centro de Biología Molecular “Severo Ochoa” (CBMSO) (CSIC-UAM), Madrid, Spain

**Keywords:** CKD, fibrosis, lipids, metabolism

The kidney is an organ that, albeit exhibiting topologic metabolic heterogeneity, derives most of its energy from oxidative metabolism. In particular, in the proximal tubular compartment, constitutive glycolysis is vestigial and the large amount of ATP required to perform its multiple transport functions is obtained through oxidative phosphorylation in the mitochondria using fatty acids as the main source of energy.^1^ Fatty acid oxidation (FAO) requires the concourse of specialized enzymes for the transport into the mitochondria of long-chain fatty acids (LCFA, more than 13 carbons) that are not freely diffusible. The limiting enzyme in this process is Carnitine palmitoyl transferase type 1 (CPT1), which, by using the carnitine shuttle, facilitates the entry of these molecules into the mitochondrial intermembrane space and ultimately into the mitochondrial matrix. *Cpt1a* is the predominant isoform in the kidney. The pathophysiologic relevance of FAO reduction in CKD has fostered the study in depth of the role of *Cpt1a* in kidney human pathology and in experimental models of chronic kidney injury.^2^

Reports from several laboratories using conditional genetic mouse models of suppression or overexpression of *Cpt1a* support that this enzyme is essential for the maintenance of a healthy mitochondrial phenotype in the proximal tubule (PT) as well as capable of conferring protection against kidney fibrosis development.^3,4^ More recently, a report from the same research group of authors as the article object of this commentary suggested that tubular *Cpt1a* deletion has only limited consequences in the context of aging and chronic kidney damage.^5^ This study showed that the conditional tubule suppression of *Cpt1a* in aged mice subjected to aristolochic acid injury or unilateral ureteral obstruction favors macrophage infiltration but has a minor repercussion in tubular injury without differences in fibrosis compared with aged wild-type animals. Interestingly, this work also pointed out an upregulation of PPARα-dependent genes and enhanced peroxisomal function, the latter mediating the oxidation of very long-chain fatty acids (more than 22 carbons) not linked to ATP production.^5^ Considering the lack of evidence of CPT1B or CPT1C isoform expression compensating the absence of *Cpt1a* in kidney tubular cells, the authors propose that the preservation of FAO at a reduced rate was accounted for only by the increased peroxisome oxidation of LCFA into medium-chain fatty acids, which can then enter the mitochondria for further metabolism. However, the relevance of peroxisomal function in injured PT warrants further investigation.

**Figure 1:**
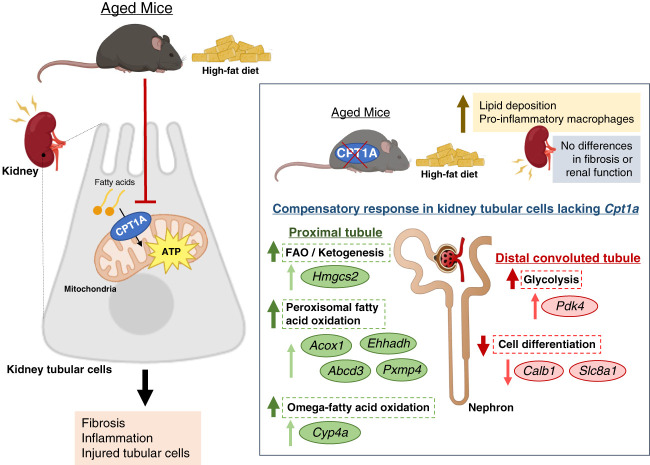
**Compensatory response in kidney tubular cells lacking *Cpt1a* in aged mice exposed to high-fat diet.** *Abcd3*, ATP-binding cassette subfamily D member 3; *Acox1*, Acyl-CoA oxidase 1; *Calb1*, calbindin 1; *Cpt1a*, carnitine palmitoyltransferase 1A; *Cyp4a*, cytochrome P450 family 4 subfamily A member 11; *Ehhadh*, enoyl-CoA hydratase and 3-hydroxyacyl CoA dehydrogenase; FAO, fatty acid oxidation; *Hmgcs2*, 3-hydroxy-3-methylglutaryl-CoA synthase 2; *Pdk4*, pyruvate dehydrogenase kinase 4; *Pxmp4*, peroxisomal membrane protein 4; *Slc8a1*, solute carrier family 8 member A1.

In their current article, Funk *et al.*^6^ explore their hypothesis in a more extreme context of metabolic stress by using kidney tubule-specific *Cpt1a*-deficient mice subjected to aging with high fat diet. Although lipid deposition and inflammation were increased in kidney tissue from animals with tubular *Cpt1a* suppression, the fibrotic phenotype was not exacerbated compared with controls (Figure 1), suggesting again that *Cpt1a* is not required to maintain healthy tubules during aging and injury. By applying single-nuclear RNA-seq transcriptomic profiling, the authors identified 16 different clusters and found that inflammation-related pathways were more conspicuous in distal convoluted tubule (DCT) compared with PT, which showed a higher degree of modified metabolic pathways. Noticeably, DCT, which had been shown to express more *Cpt1a* than PT in the previous study,^5^ also showed differential expression of genes related to morphogenesis, hinting at potential previously unknown roles of *Cpt1a* beyond FAO. In addition, this work confirmed previous results concerning the expression of genes germane to FAO and ketogenesis (*Hmgcs2*) and peroxisomal function (*Acox1*, *Ehhadh*, *Abcd3*, and *Pxmp4*) in the PT. An additional finding of interest was the upregulation of genes related to cytochrome P450 CYP4A family in PT, suggesting that omega oxidation of fatty acids, a process which fuels into peroxisomal very long-chain fatty acid oxidation, was part of the compensatory response to *Cpt1a* deletion. By contrast, the gene encoding pyruvate dehydrogenase kinase 4 was upregulated in the DCT. This enzyme inhibits pyruvate dehydrogenase shifting metabolism toward production of lactate, thus pointing to compensation of reduced mitochondrial oxidation by enhancing anaerobic glycolysis. In DCT, downregulation of genes involved in kidney development such as *Calb1*, which also resulted in a decrease of its cognate protein Calbindin 1, reinforced the hypothesis pivoting around a role for *Cpt1a* in the maintenance of a healthy differentiated state in cells pertaining to this tubular portion (Figure 1).

The two previously commented studies suggest that the kidney may compensate for the lack of *Cpt1a* and compromised mitochondrial FAO by increased peroxisomal activity to perform this function. This finding also points out divergent effects of cellular bioenergetics and lipid accumulation for different responses underlying CKD. Increased lipid accumulation was shown to contribute to tubular injury and inflammation in CKD.^7^ However, the lipotoxic effect is likely modulated by other factors as, consistently with these findings, lipid overload caused by enhanced tubular CD36 expression did not result in spontaneous kidney fibrosis in mice.^2^ To explain discrepancies with previously published work, the authors invoke differences in experimental models of damage and candidly admit potential limitations of the results related to the number of animals. In addition, future studies should formally address peroxisomal function and the other identified compensatory responses in the same context, thus providing a firm framework to interpret data on differential gene expression, and importantly, to understand to what extent these cellular adaptations counteract kidney damage. Although the suggestion of a new role for *Cpt1a* unrelated to its metabolic function, *i.e*., maintenance of a DCT differentiated phenotype is attractive, further work is necessary to explore in depth and confirm or refute this tenet.

A common finding in the phenotype of different organs where reduced FAO is present is lipid accumulation, as exemplified by studies in the liver where CPT1 was suppressed in a conditional manner.^8^ This is most likely related to an impairment of the mobilization and removal of lipid droplets (LDs), which also exhibit cellular compartmentalization, a process which determines the effectiveness of FAO and, therefore, lipotoxicity. Recent work has highlighted the relationship between CPT1 activity and mitochondrial dynamics and morphology with important consequences in FAO and gluconeogenesis in primary hepatocytes.^9^ According to these data, mitochondrial architecture would modify the sensitivity of CPT1 to malonyl-CoA, its natural allosteric inhibitor. Moreover, a recent elegant study demonstrated that the glycolytic enzyme phosphofructokinase, liver type governs lipolysis by promoting the interaction of CPT1 with the droplet-coated protein Perilipin 2 (PLIN2) and the importance of this interaction for the tethering and mobilization of LDs.^10^ Of importance, a recent comprehensive single-cell transcriptomic profiling study in a model of kidney ischemic damage identified PLIN2 as a crucial enzyme for LDs mobilization in the PT and thus for epithelial proliferation and tubule regeneration,^11^ with independence of FAO. The connection of the aforementioned pieces of information provides leverage to speculate on a different role for CPT1 beyond its canonical one in LCFA transport. This new function would encompass its capacity to mobilize LDs through its binding to PLIN2, a process also regulated by mitochondrial dynamics, which may underlie responses associated to *Cpt1a* deficiency or FAO impairment in injured kidney tubular cells. Hence, it seems that once more enzymes with a perfunctory catalytic activity linked to an established metabolic role may have other unexpected functions that future work may help to unveil.
